# An Adversarial Dual-Branch Network for Nonhomogeneous Dehazing in Tunnel Construction

**DOI:** 10.3390/s23229245

**Published:** 2023-11-17

**Authors:** Zilu Shi, Junzhou Huo, Zhichao Meng, Fan Yang, Zejiang Wang

**Affiliations:** School of Mechanical Engineering, Dalian University of Technology, Dalian 116024, China; szl_dlut@mail.dlut.edu.cn (Z.S.); mengzhichao@mail.dlut.edu.cn (Z.M.); youngfine@mail.dlut.edu.cn (F.Y.); wzj2020@mail.dlut.edu.cn (Z.W.)

**Keywords:** single image dehazing, Convolutional Neural Network (CNN), Generative Adversarial Network (GAN), knowledge transfer, attention mechanism

## Abstract

The tunnel construction area poses significant challenges for the use of vision technology due to the presence of nonhomogeneous haze fields and low-contrast targets. However, existing dehazing algorithms display weak generalization, leading to dehazing failures, incomplete dehazing, or color distortion in this scenario. Therefore, an adversarial dual-branch convolutional neural network (ADN) is proposed in this paper to deal with the above challenges. The ADN utilizes two branches of the knowledge transfer sub-network and the multi-scale dense residual sub-network to process the hazy image and then aggregate the channels. This input is then passed through a discriminator to judge true and false, motivating the network to improve performance. Additionally, a tunnel haze field simulation dataset (Tunnel-HAZE) is established based on the characteristics of nonhomogeneous dust distribution and artificial light sources in the tunnel. Comparative experiments with existing advanced dehazing algorithms indicate an improvement in both PSNR (Peak Signal-to-Noise Ratio) and SSIM (Structural Similarity) by 4.07 dB and 0.032 dB, respectively. Furthermore, a binocular measurement experiment conducted in a simulated tunnel environment demonstrated a reduction in the relative error of measurement results by 50.5% when compared to the haze image. The results demonstrate the effectiveness and application potential of the proposed method in tunnel construction.

## 1. Introduction

**Figure 1:**
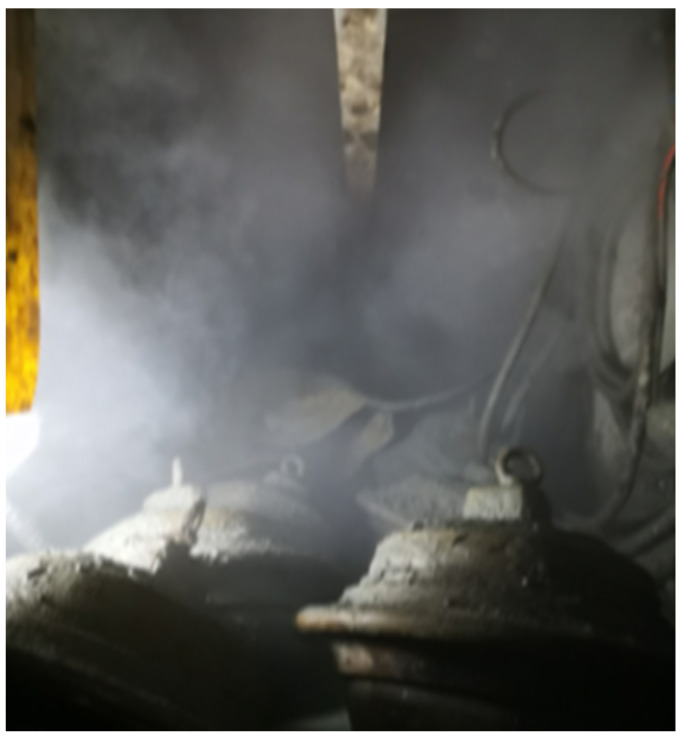
Tunnel environment.

During the construction of a tunnel using a tunnel boring machine (TBM), the rocks in contact with the tunnel’s face get crushed by the TBMs cutting head, generating a considerable amount of dust with varying particle sizes. The dust mixes with water mist via spray and ventilation dust suppression systems, forming a nonhomogeneous haze that scatters around the TBM cutter head and creates an unusual environment, as shown in Figure 1. The optical system’s image acquisition process in this environment poses some challenges, such as contrast reduction, feature blur, and color distortion. These factors impact the quality of vision-based applications that use image features in the tunnel, including object detection [1], three-dimensional reconstruction [2], and measurement [3] tasks.

Image dehazing has garnered significant attention from researchers, resulting in the proposal of several methods to address the issue. Generally, these methods fall into two categories: prior-based and deep learning-based.

Prior-based image dehazing methods utilize statistical knowledge and hand-crafted features to restore fog-free images. The Atmospheric Scattering Model (ASM) [4] was the first model proposed and estimates the transmission map by analyzing and counting image features as prior information. Dark Channel Prior (DCP) [5] extracts dark element information through local blocks to determine the transmission map but may produce estimation errors in the edges of the extracted blocks and halo artifacts in the sky and white, weakly textured areas. Recently, an improved DCP algorithm [6] based on polarized atmospheric light has been proposed, which more accurately evaluates the global ambient light and improves the image dehazing ability of the DCP algorithm. However, the polarization state estimation model used by this algorithm is not suitable for nonhomogeneous haze in tunnel construction, and the image after dehazing is dark. Haze-line [7] theory combines haze lines and boundary regularization (BR) to estimate the transmission map directly, avoiding image blocking, but may lose effectiveness in dealing with dense haze. Image Dehazing Based on Gamma Correction Prior (IDGCP) [8] uses gamma correction prior (GCP), optimizes scene albedo based on global strategy, and restores haze-free images with better color and texture detail. Image Dehazing and Exposure (IDE) [9] increases the light absorption coefficient in ASM to handle image darkness but may not perform well in nonhomogeneous haze environments. These methods may not restore images affected by dense or non-homogeneous haze, are computationally expensive, and can result in dark or low saturation problems.

Recently, deep learning approaches have shown outstanding performance in the dehazing of images. DehazeNet [10] applied a convolutional neural network to the image of dehaze for the first time, obtained a transmission map through feature extraction, multi-scale mapping, local extreme value, and nonlinear regression network structure design, and then applied an atmospheric scattering model to obtain the dehaze image. Ren et al. [11] considered the impact of different scales of information on the dehazing problem and designed a multi-scale CNN network (MSCNN), which consists of a coarse-scale network for predicting the overall transmittance map and a fine-scale network for local refinement results, and obtained a better dehazing effect. DehazeNet [10] and MSCNN [11] estimate the transmittance map and atmospheric light separately by means of ensemble learning, which is easy to cause error accumulation, cannot minimize the reconstruction loss, and its dehazing effect is limited. AOD-Net [12] is aware of this problem, so the atmospheric scattering model is changed to combine the transmittance t and the atmospheric light value A into a new variable K, and an adaptive depth model that changes with the input haze image is established. LD-Net [13] is based on the AOD-Net architecture and the dehazing model, adding a CVR module to perform pixel-level averaging and normalization of the color histogram to reduce the color distortion problem after image dehazing. These methods avoid the error caused by the manual design prior to the estimation of transmittance, but the performance in dense fog is not satisfactory. In essence, the image degradation model based on atmospheric light cannot accurately describe the imaging mechanism of complex scattering environments. Compared with the ensemble learning method using the atmospheric scattering model, the output of the network designed based on the end-to-end dehazing method is no longer the transmission map of the image to be dehazed but the direct mapping relationship from the input image to the clear image. FFA-Net [14] combines channel attention with pixel attention mechanisms and proposes an end-to-end feature fusion attention network to directly obtain dehazed images. GridDehazeNet [15] designed a multi-scale grid dehazing network based on the channel attention mechanism. It uses the residual dense block as the basic unit, which can make better use of the features learned by each convolutional layer. The above methods have achieved good evaluation scores on synthetic haze datasets, but the effect is not good when faced with nonhomogeneous dense haze images, and problems such as local haze residue and color distortion will occur.

The main contributions of this paper are as follows:

(1) A dual-branch generative adversarial network based on transfer learning is proposed to deal with the image dehazing problem of the nonhomogeneous haze field in the tunnel environment.
(2) A haze field simulation test bench is constructed that reproduces the distribution of tunnel dust and illumination, and a small-sample haze image dataset, Tunnel-HAZE, is produced in the test bench.
(3) Under the tunnel simulated fog and dust environment and dehazing conditions, the binocular vision stereo matching wear measurement of the cutter model is carried out, which proves that the proposed dehazing network has potential engineering application value in the tunnel fog and dust environment.

The rest of the article is organized as follows: Section 2 presents the network’s structure and the process of creating the dataset. Following that, Section 3 provides details on the experimental validation. Finally, Section 4 is a summary of this paper.

## 2. Methods

In this section, the dual-branch Adversarial Dehazing Network (ADN) proposed in this paper and the production of the haze image dataset Tunnel-HAZE are introduced. Firstly, the overall structure of ADN is introduced in Section 2.1. In Section 2.2, the knowledge transfer branch is introduced in detail. The multi-scale dense residual branch is then explained in Section 2.3. The loss function used in this network is described in detail in Section 2.4. In Section 2.5, the production of the haze image dataset Tunnel-HAZE is introduced.

### 2.1. Overview

This paper uses the concept of GAN [16] in the dehazing process, where a dehazing network serves as a generator that learns the distribution of haze features in hazy images and generates clear images under the supervision of real clear images. To distinguish between true and false images, the restored, clear image generated by the network and the real, clear image are inputted into a designed discriminator. This generates incentives for the generator to constantly improve its performance, thereby producing output results that are consistently closer to the distribution of real, clear images. The discriminator’s structure is similar to that in pix2pix [17], with a deeper network and a maximum number of channels extended to 1024.

**Figure 2:**
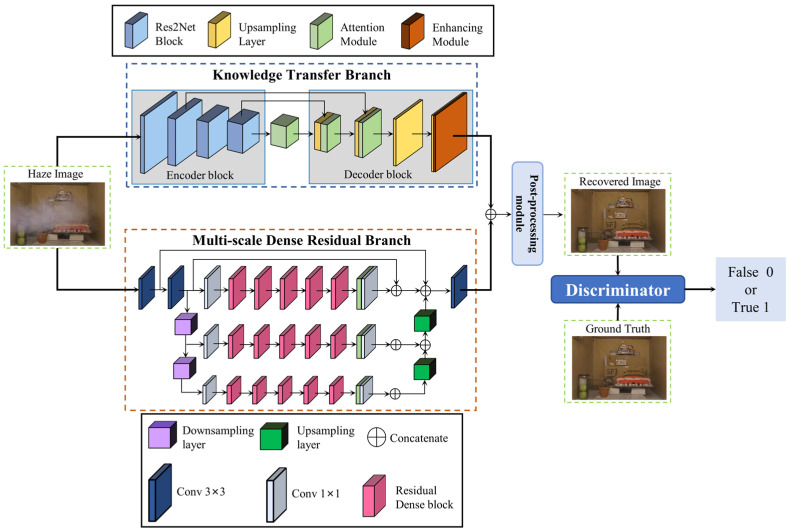
The network structure of the proposed method.

The design of a dual-branch architecture enables the use of different features from the same input, which are then combined using appropriate fusion strategies. This enhances the performance of image dehazing since it allows for comprehensive information from both branches. Figure 2 illustrates this process, where haze images pass through two sub-network branches—the knowledge transfer and multi-scale dense residual sub-networks. The resulting feature maps from each branch are then concatenated through channel concatenation. Finally, a clear image is generated through a post-processing convolution layer and tanh activation function.

The network structure of each branch is elaborated on in detail as follows.

### 2.2. Knowledge Transfer Branch

The framework for the transfer of knowledge branch has been inspired by the network model structure in KTDN [18]. The framework comprise two parts: the encoder and the decoder. The encoder is used for the extraction of image features, while the decoder includes an up-sampling layer, an attention module, and a detail enhancement module for effective image reconstruction. Moreover, a skip connection is added between the encoder and decoder to facilitate the reconstruction process by saving and transferring the features extracted by the encoder.

**Res2Net Encoder.** Res2Net [19] is a variation of the ResNet [20] architecture that utilizes grouped multi-scale convolution in the residual block, which helps to obtain fine-grained features. This architecture has proven to provide superior results for visual tasks like image classification and object detection. The encoder in this paper uses the first four parts of the Res2Net101 network structure to extract haze image features. Additionally, the pre-training parameters of Res2Net101 on ImageNet are utilized for importing the model’s training results. Using pre-trained parameters can accelerate the convergence speed of the model and reduce training costs.

**Figure 3:**
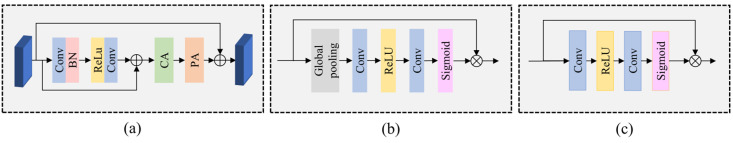
Attention module. (**a**) Feature attention module (FAM); (**b**) Channel attention block (CA); (**c**) Pixel attention block (PA).

**Feature Attention module.** The network structure incorporates channel attention inspired by SE-Block [21] to reinforce channel connections. This enables adaptive allocation of resource weights for each convolutional channel, specifically enhancing haze-related features with the highest amount of information. To tackle the nonhomogeneous distribution of fog or dust on image pixels, pixel attention is utilized. This generates distinct weights for each pixel of image features, allowing the network to concentrate on effective information such as texture, color, and haze area. Channel attention and pixel attention are combined to form the attention module, as presented in Figure 3. Channel attention is a mechanism that determines the significance of various channel feature maps in a neural network. It achieves this by assigning weights to each channel, indicating their importance. Rather than focusing on individual feature points within a channel, channel attention multiplies the respective weight with each channel’s feature map to determine varying levels of attention. The pixel attention mechanism enhances the performance of computer vision tasks by enabling the model to automatically choose pixels or areas of importance. It proves especially beneficial for managing larger images or intricate scenes, as it assists the model in concentrating on and extracting crucial information. Combining the two attention mechanisms can improve the performance of the network.

**Figure 4:**
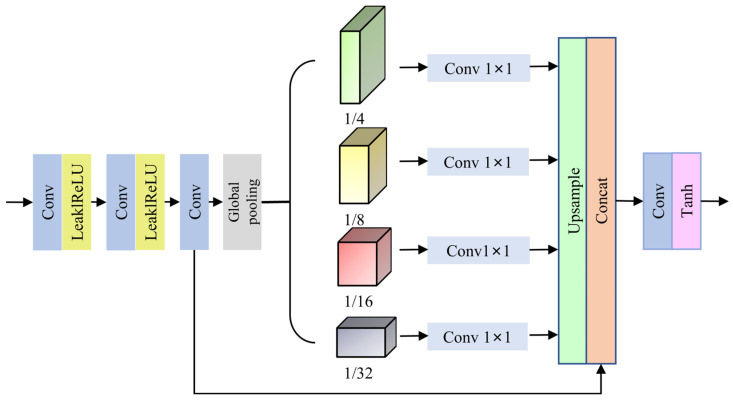
Enhanced module.

**Detail Enhancement module.** Once the haze image has been decoded, the detail enhancement module is added, as shown in Figure 4. This module is a multi-scale pyramid pooling block that subsamples the feature map to four different scales (1/4, 1/8, 1/16, and 1/32) using a global average pooling layer. By doing so, it can learn different receptive fields and restore image pixel block details and textures more effectively. After this, the multi-channel information of the feature maps at different scales is combined into a single channel using 1 × 1 convolution. Finally, the original feature maps and multi-scale feature maps are concatenated to integrate context information.

### 2.3. Multi-Scale Dense Residual Branch

In the previous section, the knowledge transfer branch primarily extracted low-frequency basic features with pre-trained weights. However, a significant amount of vital information is lost in a large range of skip connections, which is necessary for recovering local details of clear images. Relying solely on a single branch makes it arduous to deal with the problem of dehazing in complex, nonhomogeneous dehazing environments. Therefore, a multi-scale, dense residual sub-network is added in parallel. The fine image features that this part extracts can be used as supplementary information for the knowledge transfer sub-network. As a result, the network can deliver enhanced performance on small-scale datasets.

**Figure 5:**
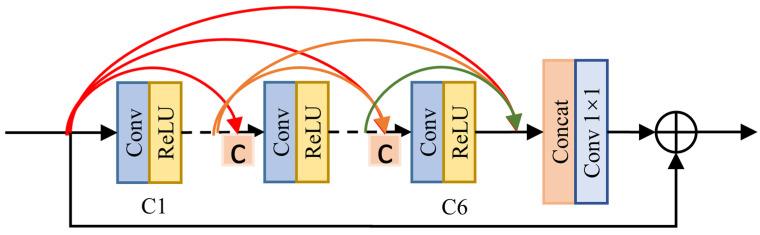
Residuals dense block.

The Residual Dense Block (RDB) has proven to be effective in generating high-quality images with enhanced resolution [22] and reducing image haze [15]. The RDB employs a dense connection that allows each convolutional layer to receive the output of the previous layer as an additional input, enabling better feature extraction and stronger connections between features at different levels. This provides more information for clear image reconstruction. After the dense connection, the channels are concatenated to retain the features adaptively. Finally, the residual connection is added to fuse local shallow information and deep information on the same feature map. This approach improves the depth of the network while maintaining convenience. Refer to Figure 5 for an illustration.

The multi-scale dense residual branch is a network that is comprised of three-channel parallel connections. The input haze image is first subjected to two convolutional layers to extract its shallow features, or what we refer to as learning inputs. The result of this process is downsampled to form three smaller branches, allowing for the learning of different scale features. Each of these smaller branches consists of five multi-scale dense residual blocks that are linked together via long-term, continuous dense connections. The number of output feature maps for each residual dense block remains unchanged. An attention module is later added at the end of each branch to adaptively assign weights to specific pixel regions as well as different feature channels of the output feature map. The next step involves utilizing the results obtained from each branch to achieve feature fusion at various levels through up-sampling and concatenation operations. This is achieved by using Pixel Shuffle, which offers better visual effects as opposed to the traditional linear interpolation method. Unlike downsampling, which typically makes use of a pooled layer, a convolutional layer is utilized to reduce information loss. Additionally, a global residual connection is included between the end of each branch and the shallow input to reduce model complexity and overfitting. The input and output channels of residual-dense blocks in the three branches are each set to 32, and the convolutional layer is configured to have six layers.

### 2.4. Loss Function

To train the network properly and guide each layer’s parameters in the right direction, we devised a loss function comprising four constituents that helps in measuring the divergence between the dehazed image generated by the network and the actual clear image. The loss function [23] is as follows:

(1) $$L=L_{1}^{S}+\alpha L_{MS\text{-}SSIM}+\beta L_{perc}+\gamma L_{adv}$$

where α=0.5; β=0.01; γ=0.005 are the hyperparameter weights for each loss function. $L_{MS\text{-}SSIM}$ is Multi-Scale Structural Similarity (MS-SSIM) loss; $L_{perc}$ is perceptual loss; $L_{adv}$ is adversarial loss.

**Smooth L1 loss.** Smooth L1 loss [24] offers the benefits of both L1 loss and L2 loss, leading to improved strength and quicker convergence. This helps to maintain a good level of influence on the brightness and color of the output image that is produced by the network.

(2) $$L_{1}^{S}=\frac{1}{N}\overset{N}{\underset{i}{\sum }}S_{1}\left(I_{gt}-f_{P}\left(I_{hazy}\right)\right)$$

(3) $$S_{1}\left(Z\right)=\{\begin{array}{c} 0.5Z^{2}\;\mathrm{if}\left|Z\right|<1\\ \left|Z\right|-0.5\;\;others\; \end{array}$$

where $I_{gt}$ and $I_{hazy}$ are real clear images, and haze images respectively; $f_{P}\left(\cdot \right)$ is the mapping function from input to output corresponding to the designed network model; *N* represents the number of pixels in the image.

**Multi-scale structural similarity loss.** To improve the dehazing results of the network in accordance with human visual perception, we implement the Multi-Scale Structural Similarity (MS-SSIM) [25] loss function during network training. This loss function takes into account the image’s resolution and helps retain high-frequency details more effectively. Let X and Y denote two windows of common size centered at pixel i in the dehazed image and the haze-free image, respectively. Use a Gaussian filter to X and Y, and compute the resulting means $\mu_{X}$, $\mu_{Y}$, standard deviations $\sigma_{X}$, $\sigma_{Y}$ and covariance $\sigma_{XY}$. The SSIM for pixel i is defined as:

(4) $$SSIM\left(X,Y\right)=\frac{2\mu_{X}\mu_{Y}+C_{1}}{\mu_{X}^{2}+\mu_{Y}^{2}+C_{1}}\cdot \frac{2\sigma_{XY}+C_{2}}{\sigma_{X}^{2}+\sigma_{Y}^{2}+C_{2}}=l\left(i\right)\cdot cs\left(i\right)$$

(5) $$L_{MS\text{-}SSIM}\left(i\right)=1-l_{M}^{\alpha }\left(i\right)\cdot \overset{\underset{M}{j=1}}{\prod }{\left[cs_{j}\left(i\right)\right]}^{\beta_{j}}$$

where *l* (i) represents luminance and *cs* (i) represents contract and structure measures, *C*_1_, *C*_2_ are two variables to stabilize the division with weak denominator.

**Perceptual loss.** Utilizing perceptual loss as a generalized structured output loss has the potential to enhance the model’s performance compared to relying solely on pixel loss. By incorporating this approach, the model can more effectively reconstruct fine details while training. This particular study employs the VGG16 network model, which was pre-trained on ImageNet [26], as the loss network. To calculate the perceptual loss, the L1 loss of this network is utilized.

(6) $$L_{\mathrm{perc}}=\frac{1}{N}\underset{i}{\sum }\frac{1}{C_{i}H_{i}W_{i}}\left|\phi_{i}\left(f_{P}\left(I_{hazy}\right)\right)-\phi_{i}\left(I_{gt}\right)\right|$$

where $C_{i},\;H_{i},\;W_{i}$ denote the channel, height, and width of the feature map in the i-th layer of the VGG16 network, and, $\phi_{i}\left(\cdot \right)$ is the activation of the i-th layer.

**Adversarial loss.** Adversarial loss [27] helps recover images with finer textures. Its loss function can be described as:

(7) $$L_{adv}=\overset{N}{\underset{n=1}{\sum }}-\log D\left(f_{P}\left(I_{hazy}\right)\right)$$

where D(⋅) is the output of the discriminator, which represents the probability that the haze-free image output by the network is considered a real, clear image.

### 2.5. Construction of the Tunnel-HAZE Dataset

The dataset used in deep learning for dehazing plays a crucial role in model training by recording the features of the scene object and the distribution information of the scattering medium. The most commonly used datasets in image dehazing include the RESIDE [28] dataset, which is a synthetic haze dataset based on atmospheric scattering models, and the O-HAZE [29] and NH-HAZE [30] datasets, which are artificial nonhomogeneous haze scene datasets featuring indoor and outdoor landscapes. These datasets feature either computer-generated haze, evenly distributed mist in real-world scenes, or haze simulated by a smoke generator. However, they may not fully reflect the characteristics of artificial light sources, poor colors, low contrast, and nonhomogeneous dust distribution in tunnel environments.

**Figure 6:**
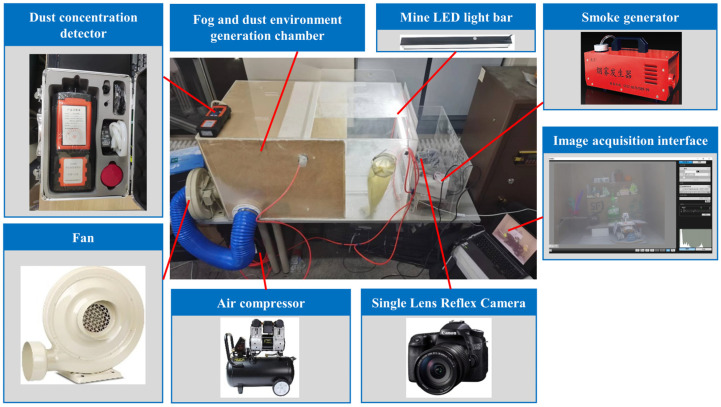
Tunnel fog and dust environment simulation experimental platform.

**Figure 7:**
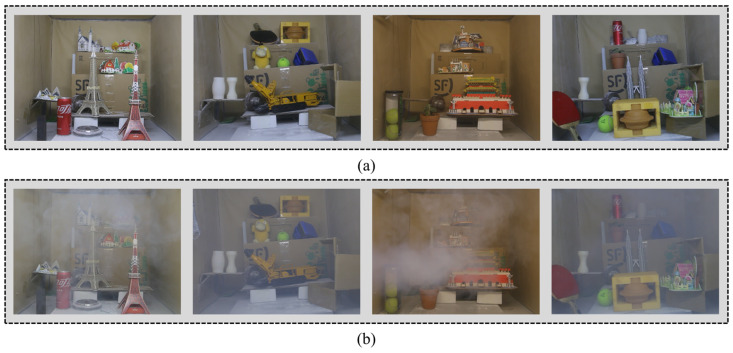
Tunnel-HAZE dataset images. (**a**) Ground truth images; (**b**) Haze images.

Based on field tests and studies, it has been observed that without dust control measures, the concentration of rock dust in TBM tunnels can reach 1000 mg/m^3^ [31], with dust particles primarily concentrated in the 1–10 μm and 50–127 μm intervals and an average particle size of 55 μm [31]. To create a dataset that simulates the tunnel haze environment, we developed a small sample dataset called Tunnel-HAZE using a tunnel environment simulation experiment platform (Figure 6). The dataset consists of 10 different scenes with varying light and scattering medium concentrations, resulting in 98 haze images and 22 corresponding clear images. Some of these images are shown in Figure 7, where the scattering medium in the hazy images is non-homogeneously distributed in the scene.

**Figure 8:**
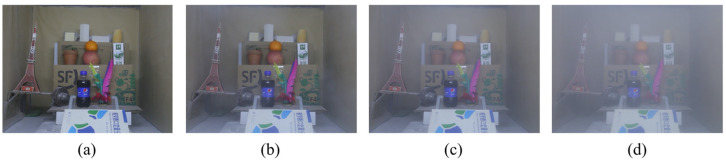
Images with different dust concentrations. (**a**) Ground truth; (**b**) Light dust; (**c**) Moderate dust; (**d**) Heavy dust.

The production of each scene is produced in the fog and dust environment generation chamber. The inner wall of the fog and dust environment generation chamber is pasted with dark, rough kraft paper to simulate the tunnel wall, and the adjustable LED mining light bar is used to simulate the real-scene lighting conditions of the tunnel. We simulated two kinds of scattering medium environments: dust and water mist. The dust environment simulation in the scene is achieved by blowing quartz, sandstone, and granite powder through air compressors, and the particle diameters are 18 μm, 38 μm, and 45 μm, respectively. The water mist environment simulation is realized by the YWQ-180 smoke generator, which atomizes water and alcohol into suspended particles with a diameter of 1–2 μm. Sufficient blending between the scattering medium and the scene and the subsequent cleaning process is achieved by adjusting the centrifugal extraction fan. We used the Canon EOS70D SLR camera and the FCJ200 light scattering dust concentration measuring instrument to simultaneously record the process of the haze environment in the scene. The shooting time was set to 3 min, and the shooting distance was between 0.8 and 1.2 m. Camera parameters are set to the same level when shooting different hazy scenes. Finally, the video is intercepted to generate 1440 × 1080 pixels of PNG-format scene images; each scene contains three different concentrations of dust environment images, as shown in Figure 8.

## 3. Experiments

In this section, the implementation details are first introduced, including the dataset introduction, training parameter settings, and experimental environment. The proposed method’s performance is then compared to other dehazing methods, both quantitatively and qualitatively. An ablation study is conducted to showcase the strengths of each component in the network. To validate the effectiveness of this method, disc cutter wear is measured in a simulated tunnel, verifying its engineering application value.

### 3.1. Implementation Details

In this paper, we train and evaluate the proposed network on synthetic and real datasets, respectively. The synthetic datasets are trained and tested using the OTS subset and the SOTS outdoor subset from the RESIDE dataset. The real dataset includes the dataset used in the NTIRE challenge and the tunnel environment simulation dataset Tunnel-HAZE produced above. The NTIRE dataset uses the NH-HAZE collection of 2020 and 2021 and contains a total of 80 pairs of pictures. A total of 70 pairs were selected for training, 5 pairs for validation, and 5 pairs for testing. In the Tunnel-HAZE dataset, 80 haze images are used for training, 10 for validation, and 10 for testing.

To reduce the demand for GPU-running cache and improve the training speed, the training set images are randomly cropped during training to obtain 256 × 256 image blocks as network input. Due to the small number of images in the Tunnel-HAZE dataset, we use the data augmentation method to randomly rotate the original image by 90°, 180°, or 270°, and flip it horizontally and vertically to increase the number and diversity of samples. During the training process, both the dehazing network and discriminator adopt the Adam optimizer, set β1 and β2 to 0.9 and 0.999, respectively, eps to 1 × 10^−8^, and the initial learning rate lr to 1 × 10^−4^. The batch size of the data are 4, the total training epoch is set to 800, and the learning rate lr is reduced by 0.5 times when the epoch reaches 200, 400, and 600. In addition, in order to maintain numerical stability in the initial stage of network training, Kaiming initialization [32] is used to initialize the learning parameters of the discriminator.

In this paper, all the experiments are conducted with a NVIDIA 3090 24 G GPU and use the PyTorch 1.7.1 (Python 3.8) framework.

### 3.2. Comparative Analysis of Results

To evaluate the performance of the proposed model more intuitively and specifically, this section compares ADN with various advanced dehazing algorithms in the same direction on the RESIDE dataset, the NH-HAZE 2020–2021 joint dataset, and the Tunnel-HAZE dataset. These algorithms are DCP [5], AOD-Net [12], FFA-Net [14], GridDehazeNet [15], and DWGAN [18]. This paper sets up three evaluation methods to evaluate the dehazing quality and efficiency of different dehazing algorithms, including qualitative visual effect comparison, quantitative results comparison, and inference time comparison.

#### 3.2.1. Qualitative Visual Effect Comparison

**Figure 9:**
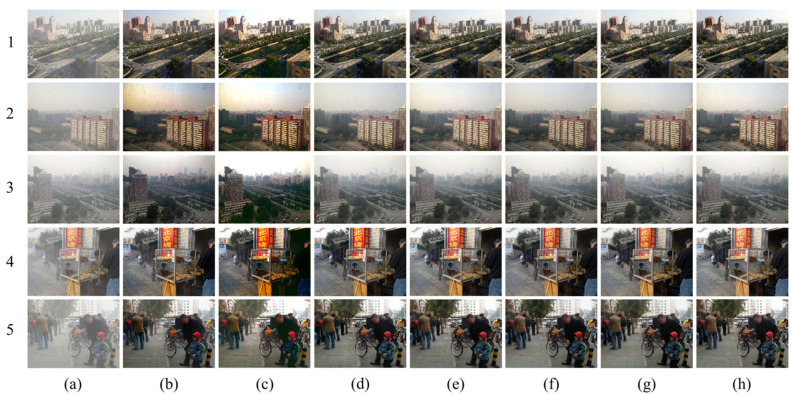
Visual comparison of qualitative results obtained by individual methods on the SOTS dataset. (**a**) Haze images; (**b**) DCP; (**c**) AOD-Net; (**d**) GridDehaze; (**e**) FFA; (**f**) DWGAN; (**g**) ADN (Ours); (**h**) Haze-free ground truth image.

**Figure 10:**
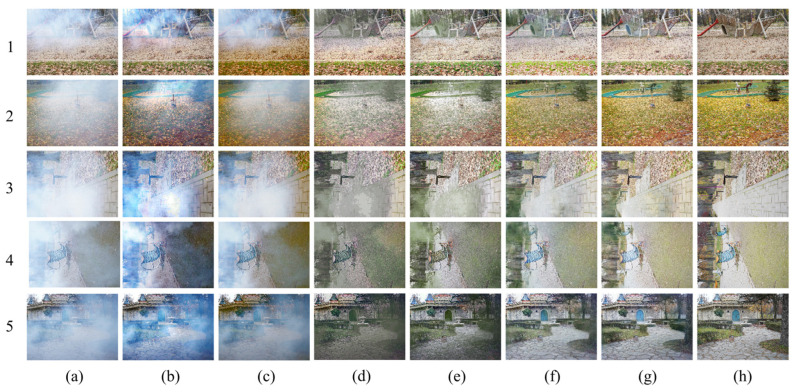
Visual comparison of qualitative results obtained by individual methods on the NH-HAZE dataset. (**a**) Haze images; (**b**) DCP; (**c**) AOD-Net; (**d**) GridDehaze; (**e**) FFA; (**f**) DWGAN; (**g**) ADN (Ours); (**h**) Haze-free ground truth image.

**Figure 11:**
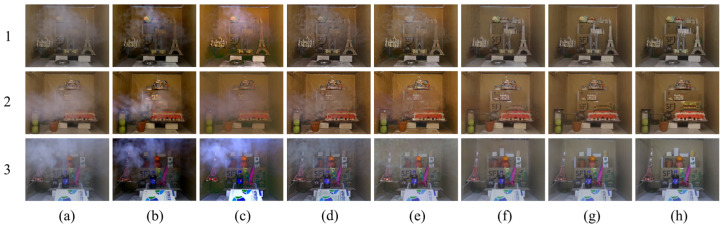
Visual comparison of qualitative results obtained by individual methods on the Tunnel-HAZE dataset. (**a**) Haze images; (**b**) DCP; (**c**) AOD-Net; (**d**) GridDehaze; (**e**) FFA; (**f**) DWGAN; (**g**) ADN (Ours); (**h**) Haze-free ground truth image.

The comparison of the qualitative visual effects of various dehazing algorithms is mainly based on the human eye’s perception of the clarity, color, and brightness of the dehazed image compared to the real clear image. Figure 9, Figure 10 and Figure 11 depict the test results of different dehazing algorithms on three datasets. Overall, the experimental methods show obvious dehazing effects when dealing with synthesizing homogeneous haze images. Physical model-based dehazing methods such as DCP and AOD-Net exhibit dehazing abilities that exceed real-clear images compared to four end-to-end deep networks. However, when dealing with real nonhomogeneous haze images in the NH-HAZE test set, DCP and AOD-Net fail first and only effectively remove some haze around the edges of the image. Only DWGAN and the proposed method maintain good dehazing ability for nonhomogeneous haze images in the Tunnel-HAZE test set.

The dehazing network proposed in this paper has achieved good visual effects on the three test sets, especially when dealing with real nonhomogeneous haze images; it has recovered more important details; and the contrast is closest to the real clear image. The brightness of the dehazed image by the DCP algorithm is dark, and severe color distortion and artifacts appear in the sky and white, weak texture areas. It is invalid for nonhomogeneous fog images, as shown in Figure 9 and Figure 10(2b). AOD-Net and DCP algorithms are similar. GridDehazeNet and FFA have similar characteristics and have a certain effect on nonhomogeneous haze images, but a large number of high-frequency details are lost in the restored clear image (the stone road lacks texture in Figure 10(3d,3e), and there are obvious color cast and color distortion problems (as shown in Figure 11d,e, the color of the two columns is yellowish, and the color of the door of the stone house in Figure 10(5d,5e) does not correspond to the ground true image). The dehazing effect of the DWGAN network is second only to the algorithm proposed in this paper, but there are obvious noise problems in the test results on the Tunnel-HAZE dataset in Figure 11.

**Figure 12:**
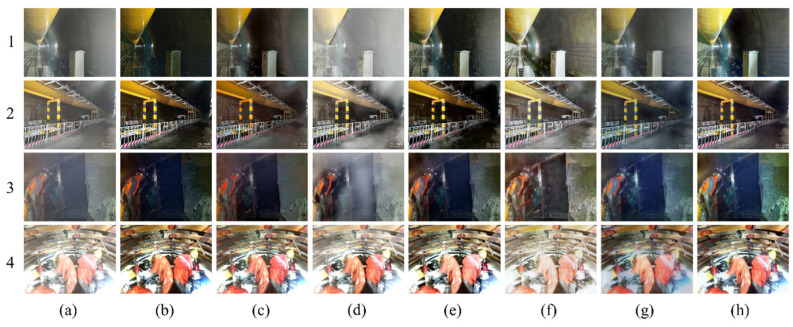
Visual comparison of qualitative results obtained by individual methods in a real tunnel environment. (**a**) Haze images; (**b**) DCP; (**c**) AOD-Net; (**d**) GridDehaze; (**e**) FFA; (**f**) DWGAN; (**g**) ADN (Ours); (**h**) Haze-free ground truth image.

We followed the experiments in DDAP [33] and conducted more tests on the methods proposed in this article. The dehazing network proposed in this paper is tested on haze images taken in a real tunnel environment. We continuously shoot at the same location to obtain hazy images and haze-free, clear images. For the first two hazy images in the tunnel, we waited for the ventilation and dehazing system in the tunnel to clear the haze before taking clear images. For the next two pictures of people, since they are in a relatively confined space, we used specific ventilation devices to remove the haze in the environment and capture clear images. The images after dehazing also show good results compared with other methods. The color and contrast of the image after dehazing are closer to the real, clear images. The dehazed image of the DCP algorithm is overall darker, especially when facing the lighting conditions in the tunnel environment. Severe dark effects appear on walls and low-brightness areas, as shown in Figure 12(1b,2b). AOD-Net has artifacts on walls and dark areas when faced with the haze images of the real tunnel environment, as shown in Figure 12(1c,2c). GridDehazeNet produced a hazy effect in some areas. The image recovered by FFA has a large area of dark blocks in the tunnel and on the ground, and many details of the image are lost, as shown in Figure 12(2e,3e). The DWGAN network generates a lot of noise and has artifacts at the wall and ground locations, as shown in Figure 12(1f,2f,4f). The method proposed in this article has better visual effects, retains more details of the image, and produces fewer artifacts.

**Figure 13:**
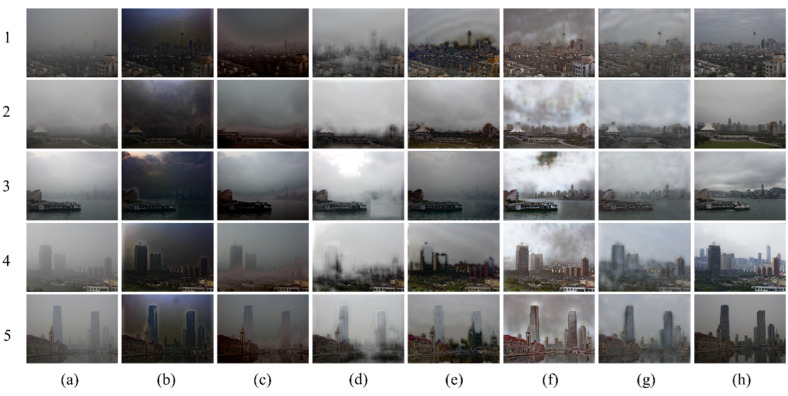
Visual comparison of qualitative results obtained by individual methods on heavy hazy images. (**a**) Haze images; (**b**) DCP; (**c**) AOD-Net; (**d**) GridDehaze; (**e**) FFA; (**f**) DWGAN; (**g**) ADN (Ours); (**h**) Haze-free ground truth image.

The proposed dehazing method is tested on real-world images with heavy haze. We used the BeDDE dataset and selected images of heavy-hazy areas. The BeDDE contains 208 image pairs collected from 23 provincial capital cities in China. For each city, one clear image and several hazy images from the same place are provided. In this step, an image of a fixed place was collected at a time between 8:00 and 9:00 each day for a period of 40 days. Such collections were conducted simultaneously at 34 provincial capitals in China in one year, and the representative scenes in those cities were chosen as the collection sites. Clear images are selected from these captured images. The method shows some dehazing effects when dealing with heavy haze, and the contrast is closest to the real, clear image. The DCP algorithm results in darker image brightness and shows less significant dehazing effects when confronted with heavy haze. AOD-Net tends to lose a considerable amount of image features while removing dense haze, as shown in Figure 13(2c,4c,5c). GridDehaze incompletely dehazes in the presence of heavy fog, exhibiting dehazing effects only in areas with less haze nearby. This method blurs the original image features and generates some white patches in certain areas, as shown in Figure 13(1d,4d,5d). FFA, when dehazing, introduces some artifacts in the sky region, as depicted in Figure 13(e1,e4,e5). Furthermore, it produces white patches in high-rise areas during dehazing, as seen in Figure 13(e4,e5). DWGAN, while dehazing, causes color distortion and introduces noticeable artifacts in the sky region, with some areas showing black spots, as shown in Figure 13(f2,f4).

#### 3.2.2. Quantitative Results Comparison

This paper evaluates different dehazing algorithms using objective indicators commonly used in image restoration, namely Peak Signal-to-Noise Ratio (PSNR) and Structural Similarity (SSIM) [34]. The PSNR ranges from 0 to 40 and the SSIM ranges from 0 to 1, with higher values indicating less difference between the dehazed and clear images. The statistical results are shown in Table 1. The method in this paper has the best values on PSNR and SSIM. It exceeds second place by 0.8 dB and 0.031 dB on the NH-HAZE dataset and by 4.07 dB and 0.032 dB on the Tunnel-HAZE dataset, which is in line with the comparative judgment of subjective visual effects. For the synthetic dataset, the performance of the network in this paper is slightly lower than that of the FFA dehazing network in terms of the PSNR metric. The proposed method in this paper outperforms other methods for capturing hazy images in a real tunnel environment. It achieves a 2.92 dB higher PSNR value compared to the second-place method and a 0.017 dB higher SSIM value compared to the second-place method. When faced with real-world hazy images with heavy haze, the proposed method in this paper achieves a 1.64 dB higher PSNR value compared to the second-place method and a 0.063 dB higher SSIM value compared to the second-place method.

#### 3.2.3. Inference Time Comparison

We conducted a comparison of the running times of various algorithms on the same hardware using an 800 × 600 pixel test image to quantitatively assess their efficiency. The results of this comparison are presented in Table 2.

Compared to other algorithms listed, the proposed algorithm has the largest parameter scale but ranks only better than FFA-Net in terms of single image running speed. There are two branches in the algorithm, with branch one parameters accounting for 93.9% of the final method and branch two accounting for 83.2% of the whole algorithm, despite its parameter scale being at a lower proportion. The dense connections in the RDB block generate high computing consumption, leading to longer run times per image. Overall, the algorithm improves network performance at the cost of a certain operating efficiency. The single image running time is 0.147 s, which falls within the acceptable range.

### 3.3. Ablation Study

This section analyzes and evaluates the importance of two branch structures, namely transfer learning and adversarial learning modules, in the proposed network model training. This study compares the training results of four models on the Tunnel-HAZE dataset: a single knowledge transfer branch model, a single knowledge transfer branch model without pre-training model parameter import, a network model without adversarial modules, and the final complete network model. The comparison is conducted horizontally.

**Figure 14:**
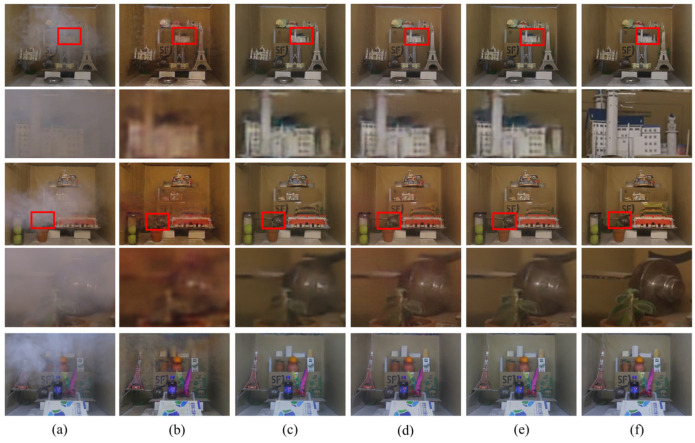
Results of the ablation experiment. (**a**) Haze images; (**b**) Branch I; (**c**) Pre-training branch I; (**d**) Dual-branch without GAN; (**e**) AND (Ours); (**f**) Haze-free ground truth image.

From the comparison results of Figure 14 and Table 3, it can be seen that branch I with pre-training parameters in ImageNet has a significant improvement in the quality of the dehazing image compared with Branch I using Kaiming initialization, and the PSNR and SSIM are also improved correspondingly. Comparing the results of the dual-branch network with the results of the single-branch network, it can be found that the dual-branch network structure has a better effect on the recovery and clarity of the local details of the haze map, and it is also reflected in the evaluation index, indicating the superiority of the dual-branch network structure. Finally, the comparison of the locally enlarged images in columns 3(d) and 3(e) proves that the images recovered by the GAN dual-branch network have sharper edge contours and color fidelity.

### 3.4. Measurement of Cutter Wear

**Figure 15:**
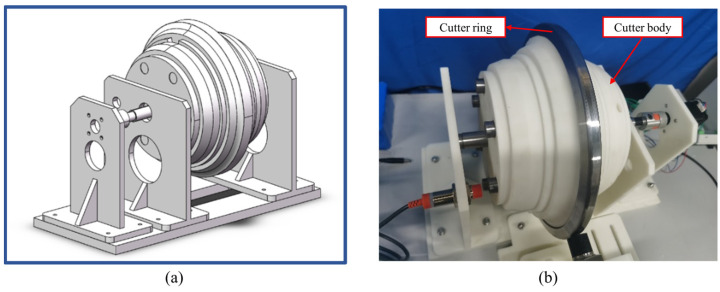
Cutter model. (**a**) A three-dimensional model; (**b**) Physical object.

For the experiment, a 17 inch disc cutter was chosen as the subject of measurement, and a cutter model was designed on a 1:2 scale. To emulate different levels of wear on the cutter rings under actual conditions, the measurement involved assembling cutter rings with various outer diameters on the cutter body. The cutter ring was made of metal material, and the entire cutter model was produced via 3D printing. The three-dimensional model and the physical object of the cutter model are depicted in Figure 15.

**Figure 16:**
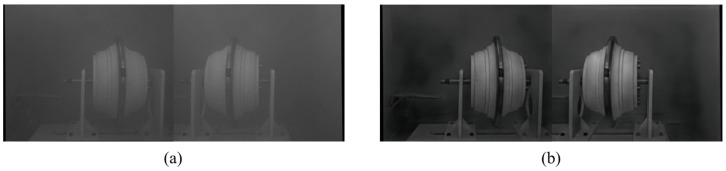
Cutter images. (**a**) Cutter haze image; (**b**) Cutter dehazing image.

Once the cutter model is completed, constructing the visual shooting, image processing, and environment simulation systems is conducted in the same manner as described in Section 2.5. The proposed dual-branch adversarial dehazing network was utilized to dehaze the haze image of the cutter. The model was trained on the Tunnel-HAZE dataset. The mass concentration of the haze measured 936 mg/m^3^, indicating that it belonged to the category of dense haze. The outcome of the dehazing process is presented in Figure 16. The hazy contour features of the cutter surface captured in the original image were clearly restored after the dehazing process. Additionally, some of the obscured objects that were present behind the cutter were also partially restored.

**Figure 17:**
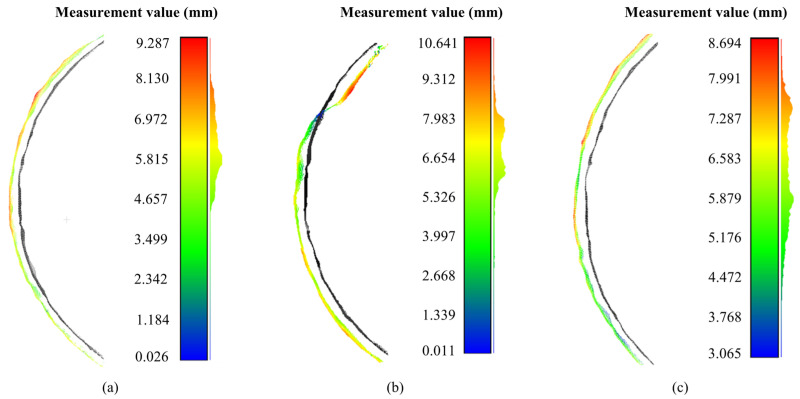
Cutter point cloud 6 mm wear measurement. (**a**) Clear image of wear measurement; (**b**) Haze image of wear measurement; (**c**) Dehazing image of wear measurement.

The trained RAFT-Stereo [35] is fed with a cutter image pair to generate a disparity map after stereo rectification. Then, using the disparity map, the reprojection matrix, and the corrected image of the left camera as a mask, the 3D point cloud of the cutter is reconstructed. Figure 17 shows the point cloud measurement results (6 mm) of the outer edge wear of the cutter ring under three different environments. The radius difference between the least square cylindrical surfaces fitted by the two measuring tool rings is taken as the measured value. The measured averages and the comparison with the real values are shown in Table 4.

The true amount of wear of the cutter rings is determined by the difference in their radius, as shown in Table 4. When the actual wear amount was 6 mm, measurements were taken in a haze-free environment and produced an average value of 6.127 mm with a standard deviation of 1.059 mm, resulting in a relative error of 2.1% compared to the true value. In a foggy and dusty environment, the average measurement value of the point cloud was 6.589 mm with a standard deviation of 1.598 mm, resulting in a relative error of 9.8%. After applying the dehazing network designed in this paper, the average measured value of the point cloud was 6.305 mm with a standard deviation of 1.091 mm, resulting in a relative error of 4.9%. When the actual wear amount was 8 mm, measurements were taken in a haze-free environment and produced an average value of 8.145 mm with a standard deviation of 1.075 mm, resulting in a relative error of 1.8% compared to the true value. In a foggy and dusty environment, the average measurement value of the point cloud was 8.730 mm with a standard deviation of 1.617 mm, resulting in a relative error of 9.1%. After applying the dehazing network, the average measured value of the point cloud was 8.361 mm with a standard deviation of 1.112 mm, resulting in a relative error of 4.5%. Overall, under a haze-free environment, the point cloud measurement accuracy was 98.0%, indicating high reliability in the binocular vision measurement method. The dehazing process improved measurement accuracy by reducing the average relative error by 4.7% and reducing the standard deviation by an average of 0.507 mm. This improvement suggests that the surface flatness of the reconstructed point cloud has been improved. The dehazing process effectively restores some obscured and damaged image features, which helped to improve the quality of the 3D reconstruction point cloud.

## 4. Conclusions

This paper introduces a new method to address the problem of image dehazing in nonhomogeneous scattering environments in tunnels. The proposed method is a dual-branch dehazing network based on GAN. It uses a knowledge transfer branch and a multi-scale dense residual branch in parallel to restore the haze map in tunnel environments. In addition, a dataset called Tunnel-HAZE to support tunnel scene dehazing is made. Comparing the results with existing methods, the proposed dehazing network is found to be more effective in this application and can improve the robustness of tunnel vision systems.

Due to a lack of identifiable data about the subject, the algorithm falls short of achieving optimal results for restoring images entirely engulfed in thick fog. Future research directions will mainly focus on meeting the demands of real-time dehazing and improving the efficiency of the algorithm.

## Figures and Tables

**Table 1:** Evaluation index of the dehazing effect.

	RESIDE-OTS		NH-HAZE		Tunnel-HAZE		Real Tunnel Environment		Heavy Hazy Images	
	PSNR	SSIM	PSNR	SSIM	PSNR	SSIM	PSNR	SSIM	PSNR	SSIM
DCP	16.93	0.823	11.56	0.592	16.41	0.685	12.93	0.387	11.94	0.510
AOD-Net	19.99	0.818	13.03	0.622	19.38	0.783	14.50	0.342	16.71	0.714
GridDehaze	28.49	0.932	16.35	0.674	22.40	0.841	12.14	0.319	13.96	0.761
FFA	30.56	0.933	17.52	0.772	22.12	0.813	13.25	0.419	12.64	0.651
DWGAN	28.04	0.940	20.17	0.790	26.18	0.885	13.11	0.506	15.82	0.729
ADN (ours)	28.94	0.952	20.97	0.821	30.25	0.917	16.03	0.523	17.46	0.792

**Table 2:** Run-time comparison.

	AOD-Net	FFA-Net	GridDehaze	DWGAN	ADN (Ours)	Branch I	Branch II
Inference Time	0.017 s	0.378 s	0.078 s	0.095 s	0.147 s	—	—
Model Parameters	6345	4.4 M	2 M	51.5 M	52.5 M	49.3 M	3.2 M
FLOPs (×109)	3.0	2100	360	250	890	160	740

**Table 3:** Effect of different model structures on performance.

	Pre-Training	Discriminator	PSNR	SSIM
Branch I	—	√	22.45	0.788
Branch I	√	√	28.28	0.886
Dual-branch	√	—	28.36	0.905
Dual-branch	√	√	30.25	0.917

**Table 4:** Cutter point cloud wear measurement results.

Point Cloud	True Wear/mm	Measured Mean/mm	Standard Deviation/mm	Relative Error	Population Mean Error
clear image	6.000	6.127	1.059	2.1%	2.0%
	8.000	8.145	1.075	1.8%	
haze image	6.000	6.589	1.598	9.8%	9.5%
	8.000	8.730	1.617	9.1%	
dehazing image	6.000	6.295	1.091	4.9%	4.7%
	8.000	8.361	1.112	4.5%	

## Data Availability

The data that supports the findings of this study are available upon reasonable request from the authors.
